# Polysaccharides from *Vaccaria segetalis* seeds reduce urinary tract infections by inhibiting the adhesion and invasion abilities of uropathogenic *Escherichia coli*

**DOI:** 10.3389/fcimb.2022.1004751

**Published:** 2022-11-23

**Authors:** Rongmei Yao, Xin Mao, Yingli Xu, Xue Qiu, Lirun Zhou, Yaxin Wang, Bo Pang, Mengping Chen, Shan Cao, Lei Bao, Yanyan Bao, Shanshan Guo, Limin Hu, Haijiang Zhang, Xiaolan Cui

**Affiliations:** ^1^ Institute of Chinese Materia Medica, China Academy of Chinese Medical Sciences, Beijing, China; ^2^ Institute of Traditional Chinese Medicine, Tianjin University of Traditional Chinese Medicine, Tianjin, China; ^3^ Guangzhou Baiyunshan Xingqun Pharmaceutical Co., Ltd, Guangzhou, China; ^4^ Jiangsu Key Laboratory of Regional Resource Exploitation and Medicinal Research, Huaiyin Institute of Technology, Huai’an, China

**Keywords:** *Vaccaria segetalis*, urinary tract infection, uropathogenic *Escherichia coli*, adhesion, motility

## Abstract

The seeds of *Vaccaria segetalis* (Neck.) are from a traditional medicinal plant Garcke, also called Wang-Bu-Liu-Xing in China. According to the Chinese Pharmacopoeia, the seeds of *V. segetalis* can be used for treating urinary system diseases. This study was designed to investigate the underlying mechanism of VSP (polysaccharides from *Vaccaria segetalis*) against urinary tract infections caused by uropathogenic *Escherichia coli* (UPEC). Here, both *in vitro* and *in vivo* infection models were established with the UPEC strain CFT073. Bacterial adhesion and invasion into bladder epithelial cells were analyzed. We found that VSP reduced the adhesion of UPEC to the host by inhibiting the expression of bacterial hair follicle adhesion genes. VSP also reduced the invasion of UPEC by regulating the uroplakins and Toll-like receptors of host epithelial cells. In addition, the swarming motility and flagella-mediated motility genes flhC, flhD and Flic of UPEC were diminished after VSP intervention. Taken together, our findings reveal a possible mechanism by which VSP interferes with the adhesion and invasion of UPEC.

## 1 Introduction

Urinary tract infections (UTIs) are inflammatory reactions caused by bacteria and fungi that invade the urinary tract mucosa or tissues. The UTI incidence rate is between 150 and 250 million cases worldwide per year (Kyriakides et al., 2020; van Nieuwkoop, 2022). The high incidence and increasing resistance to antibacterial drugs of UTIs are significant burdens on healthcare systems. The main pathogenic bacteria of UTIs include *Escherichia coli*, *Klebsiella pneumoniae*, *Staphylococcus saprophyticus* and *Enterococcus faecalis*, among which *Escherichia coli* account for more than 80% of the total cases (Schüroff et al., 2021; Chenoweth, 2021; Patel et al., 2022). The *Escherichia coli* that cause UTIs in humans is called uropathogenic *Escherichia coli* (UPEC). The physiological process of UPEC in the host can be roughly divided into four steps: the bacteria adhere to urothelial cells, the bacteria invade the cells, the bacteria proliferate inside the cells, and the cells are broken to release the bacteria (Flores-Mireles et al., 2015). Bacterial adhesion to urothelial cells is a key link in starting the entire physiological process of urinary tract infections (Flores-Mireles et al., 2015; Das, 2020).

The high incidence of recurrent UTIs indicates that antibiotics may not be the optimal treatment for urinary tract infections. The adhesion and subsequent invasion of bacteria are critical pathogenic factors of UTIs. Recognizing specific glycoconjugate receptors on host cells through high-affinity carbohydrate antagonists and blocking FimH- and PapG-mediated bacterial adhesion to urothelial cells has become a topic of interest for researchers (Mousavifar et al., 2018; Gupta et al., 2021; Montes-Robledo et al., 2021). Researchers can develop new effective drugs against these pathogenic factors to prevent the adhesion and colonization of urinary tract pathogens in the urinary tract, which would solve the current predicament of UTI prevention and treatment.

The seeds of *Vaccaria segetalis* have been used to treat urinary system diseases for more than 2,000 years. Based on its traditional usage, we investigated the inhibitory effect of a polysaccharide extract of *V. segetalis* seeds (VSP) on the adhesion and invasion of UPEC. This paper aims to advance the scientific knowledge on this topic to find new ways and effective drugs to prevent and treat UTIs, explore the process of bacteria infecting the host, and inhibit the infection of UPEC by interfering with the bacteria-host reaction process.

## 2 Materials and methods

### 2.1 Biosafety statement

All the experiments involving live UPEC were performed in the Animal Biosafety Level 2 Laboratory (ABSL-2) at the Institute of Chinese Materia Medica, China Academy of Chinese Medical Sciences.

### 2.2 Preparation of the VSP

The *V. segetalis* seeds were provided by the Anguo herb market, Hebei Province, China. The material complied with the specification of the Pharmacopoeia of the People’s Republic of China (2015). The voucher specimen was retained in an ABSL-2 Biosafety Laboratory, Institute Chinese Materia Medica China Academy of Chinese Medical Sciences, Beijing, China (No. 201707001).

Five hundred grams of *V. segetalis* seeds were powdered and extracted with deionized water (12.5 L) for 5 hours at 95°C. The extract solution was centrifuged, and the supernatant was concentrated under a vacuum. The concentrated solution was precipitated using 80% ethanol and incubated overnight. The precipitate was dried under a vacuum to obtain the VSP. The total polysaccharide content of the VSP was determined to be 87.50% (w/w) with the phenol−sulfuric acid method (Mao et al., 2020).

### 2.3 Bacterial strain

The UPEC CFT073 strain (ATCC^®^700928™) was provided by the American Type Culture Collection. The CFT073 bacteria was statically grown in Luria-Bertani broth (Solarbio Science & Technology, Beijing, China) for 17 hours before infection. The concentration of the microbial suspension was determined by measuring the absorbance at 600 nm.

## 3 Infection and treatment

### 3.1 The 1-cell infection model

#### 3.1.1 The 1-Cell culture

The human bladder epithelial cell Line 5637 (HTB-9) was obtained from Bnbio Tech Co., Ltd, China, and cultured in an RPMI 1640 medium (8117044, Gibco, New York, USA) supplemented with 10% foetal bovine serum (35081006, CORNING, New York, USA) at 37°C in the presence of 5% CO_2_.

##### 3.1.1.1 Bacterial adhesion and invasion assays

5637 (HTB-9) cells (1×10^5^ cells/well) were seeded into a 12-well plate overnight. A bacterial control group (model group) and the VSP treatment group were used in this experiment. Before infection, 5637 (HTB-9) cells in the VSP treatment group were treated with VSP (50 μg/mL, 2 mL/well) for 24 hours. The cells of the model group were treated with a culture medium (2 mL/well) for 24 hours. After the VSP intervention, each group was incubated with CFT073 for 2 hours. The bacterial/cell ratio was 10:1.

###### 3.1.1.1.1 The bacterial adhesion experiment.

5637 (HTB-9) cells were infected with CFT073 for 2 hours, and the cells were washed three times with PBS to remove nonadherent bacteria. The cells were lysed with 0.1% sodium deoxycholate to collect the surrounding bacteria. The lysate was serially diluted with PBS, spread on MacConkey agar plates (Thermo Fisher Scientific, Massachusetts, USA) and then incubated at 37°C. The colony-forming units (CFU) were enumerated after 24 hours of culture. The adhesion rate was calculated by the number of bacteria attached to the cells relative to the total bacteria from the same experiment (Shen et al., 2014).

###### 3.1.1.1.2 The bacterial invasion experiment.

After being infected with CFT073 for 2 hours, the 5637 (HTB-9) cells were washed three times with PBS and treated with gentamicin (50 μg/mL. G8179, Solarbio, Beijing, China) for 1 hour to inactivate the extracellular bacteria. The cells were washed 3 times with PBS to remove extracellular antibiotics. The cells were then lysed with 0.1% sodium deoxycholate. The lysate was serially diluted with PBS and spread on MacConkey agar plates. After 24 hours of incubation, the number of bacterial colonies was counted, and the bacteria in the cells were quantified. The invasion rate was calculated by the number of bacteria in the cell relative to the total bacteria from the same experiment (Shen et al., 2014).

##### 3.1.1.2 Pilus adhesin gene detection

The 5637 (HTB-9) cells (1×10^5^ cells/well) were seeded into 6-well plates overnight. The UPEC CFT073 bacteria infected the 5637 (HTB-9) cells after being cultured for 24 hours. The bacteria were then added to the cell culture with a ratio of 10:1, the culture solution was aspirated after 2 hours and then treated with VSP (50 μg/mL). The model control group was made with an equal volume of the cell culture solution and incubated in an incubator at 37°C in the presence of 5% CO_2_ for 24 hours.

Total RNA was extracted from the infected 5637 (HTB-9) cells using a TRIzol reagent (Invitrogen, Massachusetts, USA). 16S rRNA was used as the internal control. The mRNA expression levels of the target genes were analysed using a One Step TB Prime Script RT-PCR Kit II (TaKaRa, Beijing, China) with an ABI 7500 (Applied Biosystems, Massachusetts, USA). And the method was as follows: 42°C for 5 min and 40 cycles of 95°C for 10 s and 60°C for 30 s. The real-time data were calculated using the 2^−ΔΔCt^ method. The primer sequences are listed in **Table 1**.

**Table 1:** Sequence of the primers used in this study.

Primer name	Source	Forwards primer	Reverse primer
FimH	*Escherichia coli*	GTACCAGCCGCCGTAATCAT	GTCGATGGCGGGTCAAGTAT
FimA	*Escherichia coli*	ACTCTGGCAATCGTTGTTCTGTCG	ATCAACAGAGCCTGCATCAACTGC
FimE	*Escherichia coli*	GCCCACTGAAGAACGGATTTT	AGTCCGGTCAGCCCTTT
CsgA	*Escherichia coli*	AGAAGGAGATATAACTATGTCTGAACT GAACATCTACCAGTACGGTGGTGGTA	GTGGTGGTGATGGTGATGGCCGTAC TGGTGCGCGGTCGCGTTGTTACCGAA
PapG	*Escherichia coli*	AGAAGGAGATATAACTATGTCTCTGGG TAACGTTAACTCTTACCAGGGTGGTAA	GTGGTGGTGATGGTGATGGCCCGGCAG GATCATCAGCAGGGTCGCAGAACCAG
16s-rRNA	*Escherichia coli*	CCCTCTACGAGACTCAAGC	TTACCCGCAGAAGAAGCA
FliC	*Escherichia coli*	ACAGCCTCTCGCTGATCACTCAAA	GCGCTGTTAATACGCAAGCCAGAA
FlhD	*Escherichia coli*	CCGAGACGAAACATAGCGGA	TGCATACCTCCGAGTTGCTG
FlhC	*Escherichia coli*	TTGTGGGCACTGTTCAAGGT	CATGCTGCCATTCTCAACCG
TLR4	mouse	CAACCTCCCCTTCTCAACCAA	AGGACAATGAACACCTCACCT
TLR5	mouse	ATTGCCAATATCCAGGATGC	CACCACCATGATGAGAGCAC
MyD88	mouse	GGTAGACCCACGAGTCCG	CACTCGCATGTTGAGAGCAG
β-actin	mouse	TATCCTGGCCTCACTGTCCA	AAGGGTGTAAAACGCAGCTC

##### 3.1.1.3 TLR gene detection

RNA was extracted to detect the expression of host Toll-like receptors (TLRs) and adaptor proteins related to bacterial adhesion. The experimental method was the same as the “2.3.1.3. Pilus adhesin gene detection”. β-actin was used as the internal control. Primer sequences are listed in **Table 1**.

##### 3.1.1.4 Bacterial motility assay

Soft agar plates (1% tryptone, 0.5% NaCl and 0.5% agar), as previously described (Cooper et al., 2012), were used to evaluate the motility of the bacteria. The UPEC strain CFT073 was cultured in an LB medium at 37°C for 17 hours and the optical density was measured at 600 nm with a UV spectrophotometer. After the bacterial solution was diluted (10^6^ CFU/mL), the VSP (50 μg/mL) was added. The same volume of normal saline was added to the model group. A sterile inoculating needle was used to pierce the culture into the middle of a soft agar plate and touching the bottom of the plate was avoided. The motility diameters were measured after 2, 6, and 17 hours of 37°C incubation. This assay was repeated six times.

##### 3.1.1.5 Flagellar motor gene detection

RNA was extracted to detect the expression of fliC, flhC, and flhD, which are related to bacterial motility. The detection method was the same as the “2.3.1.3. Pilus adhesin gene detection”. 16S rRNA was used as the internal control. Primer sequences are given in **Table 1**.

#### 3.1.2 Animal infection model

##### 3.1.2.1 Animals

The C3H/HeN mice (female, specific pathogen-free, 18~20 g) were purchased from Charles River Laboratories China (Beijing, China). All animals were maintained in a standard laboratory conditioned at a temperature of 25 ± 2°C with 50~55% relative humidity and a 12-hour light/dark cycle.

##### 3.1.2.2 Experimental design

The C3H/HeN mice were orally administered the VSP (800 mg/kg/d and 400 mg/kg/d) for three days. One hour after the last treatment, the mice were anaesthetized with isoflurane (Yuyan Instruments, Shanghai, China) and inoculated transurethrally with 50 μL of CFT073 at 2×10^7^ colony-forming units (CFUs) per mouse. Mice were sacrificed 6 hours after infection, and then the bladders were aseptically harvested.

**Figure 1:**
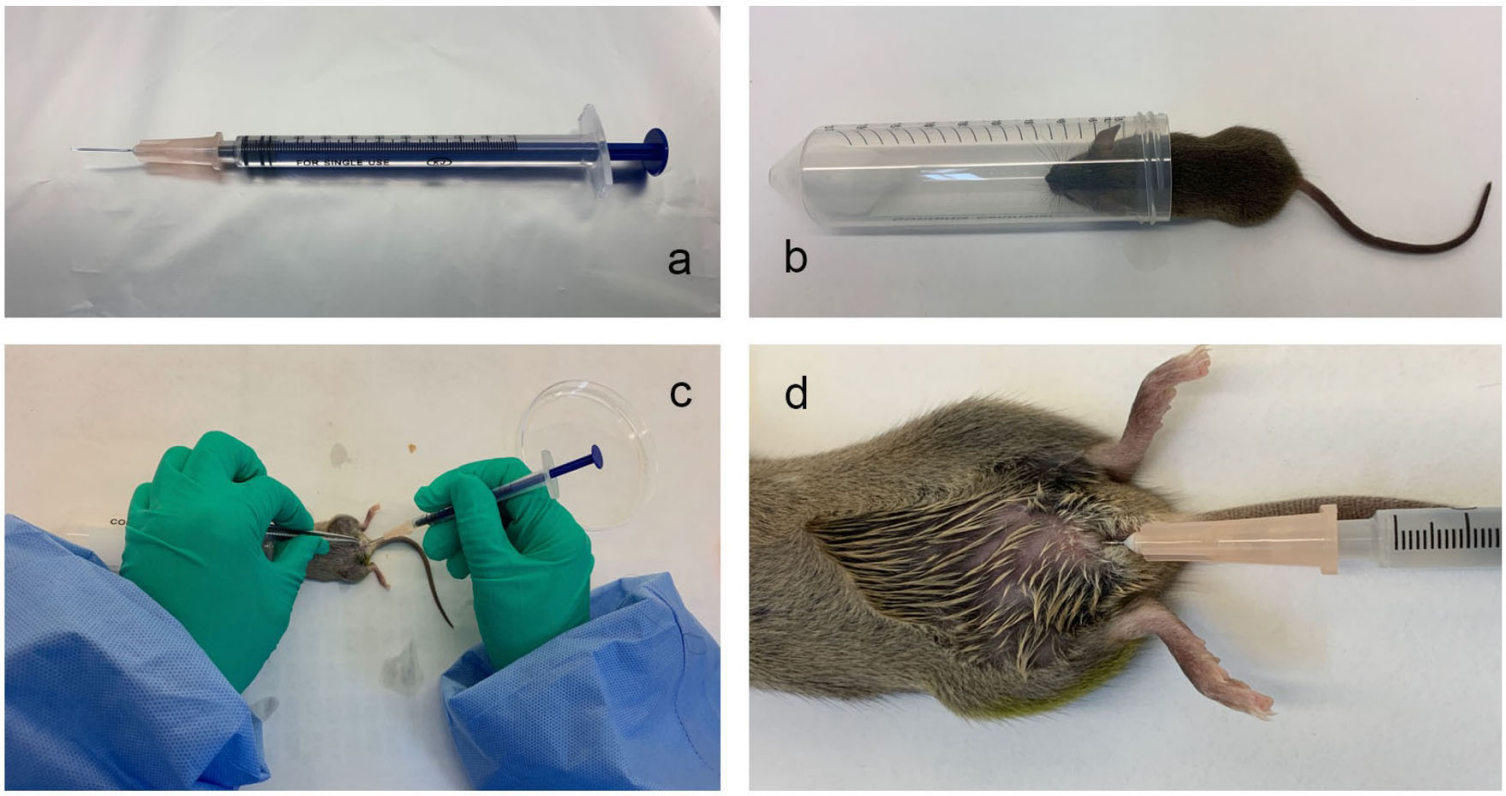
The mouse urinary tract infection model established by transurethral intravesical instillation of UPEC CFT073. **(A)**, The bladder injection device; **(B)**, The anaesthesia device; **(C, D)**, The operation method for the transurethral infusion of the bacterial fluid.

The bladder injection device: A 30-G hypodermic needle (BD Company, USA) was placed, and a soft polyethene catheter (0.61 mm outer diameter, BD Company, USA) was inserted outside the needle. The soft polyethene catheter was approximately 2-3 mm longer than the needle tip. The needle was attached to a sterile single-use syringe, as shown in **Figure 1A**.

The anaesthesia device: A 50 mL centrifuge tube was taken, a dry cotton ball was placed at the bottom of the tube, and 1 mL of isoflurane liquid (2564852, Yuyan, Shanghai, China) was soaked in the dry cotton ball, as shown in **Figure 1B**.

The Model Method: The abdomen of the mice was gently pressed with fingers to expel urine from the bladder. The mice were briefly anaesthetized with isoflurane. Medical alcohol (75%) was used to disinfect the urethral orifice and the surrounding area. The urethral orifice was gently fixed with forceps, and the bladder injection device with the nested catheter was inserted vertically into the urethral orifice and then slowly rotated. The syringe guided the injection device through the folded tissue in the urethra to the bladder (approximately 1 cm into the bladder) and then injected the diluted bacterial solution into the bladder. The perfusion volume of the bacterial solution was 50 μL/mouse, and the concentration of the bacterial solution was 2×10^7^ CFU. (**Figures 1C, D** **)**. The mice in the normal control group were perfused with saline through the urethra under the same conditions.

##### 3.1.2.3 Bacterial titer determination

The bladders from the mice were incubated in gentamicin (100 μg/mL) for 60 min and washed three times with PBS. The bladders were then homogenized in PBS (500 μL PBS/bladder), serially diluted with PBS, and plated onto MacConkey agar (200 μL homogenate/culture dish; CM1169, OXOID UK) to enumerate the intracellular bacteria after 24 hours of culture.

##### 3.1.2.4 Detection of uroplakin expression

Western blotting was employed to detect the expression of uroplakins. The mouse bladders were homogenized with a RIPA lysis buffer (Beyotime, Shanghai, China), and protein concentrations were quantified using a BCA protein assay kit (Beyotime, Shanghai, China). Equal amounts of proteins were separated by 10% denaturing SDS-PAGE gels and transferred to PVDF membranes (Merck Millipore, Massachusetts, USA). After blocking, the membranes were incubated overnight at 4°C with primary antibodies against UP Ia (ab185970, Abcam, Cambridge MA, USA), UP III (ab78196, Abcam, Cambridge MA, USA), and GAPDH (ab181602, Abcam, Cambridge, MA, USA) and then incubated with a secondary antibody (66009-1, Proteintech, Chicago, USA) for 1 hour at room temperature. Signals were visualized using FluorChem FC3 (Protein SimplIae, California, USA).

##### 3.1.2.5 Detect the position and intensity of uroplakins

Immunofluorescence was employed to detect the position and intensity of the uroplakins. The bladder tissues were fixed in 4% paraformaldehyde overnight and then dehydrated sequentially in 10%~30% sucrose solution at 4°C overnight. The samples were embedded into the optimal cutting temperature compound (Sakura Finetek, Torrance CA, USA). The sections were prepared with citrate and then blocked with bovine serum albumin at room temperature for 2 hours. The samples were incubated with primary antibodies against UP Ia (ab185970, Abcam, Cambridge MA, USA) and UP III (ab78196, Abcam, Cambridge MA, USA) at 4°C overnight and then incubated with a secondary antibody (goat anti-mouse IgG) (GB25301, Servicebio, Wuhan, China) at RT for 1 hour. The nuclei were stained with DAPI (G1012, Servicebio, Wuhan, China). Fluorescence microphotographs were captured by a Pannoramic MIDI/P250 (3DHISTECH, Budapest, Hungary).

### Statistical analyses

Statistical analyses were performed using GraphPad Prism version 6.0 (GraphPad Software, La Jolla, CA, United States). All data are presented as the means ± standard errors of the means (SEM) or means ± standard deviations (SD). Differences with *p*-values less than 0.05 were considered statistically significant.

## 4 Results

### 4.1 VSP inhibits adhesion and invasion of UPEC

**Figure 2:**
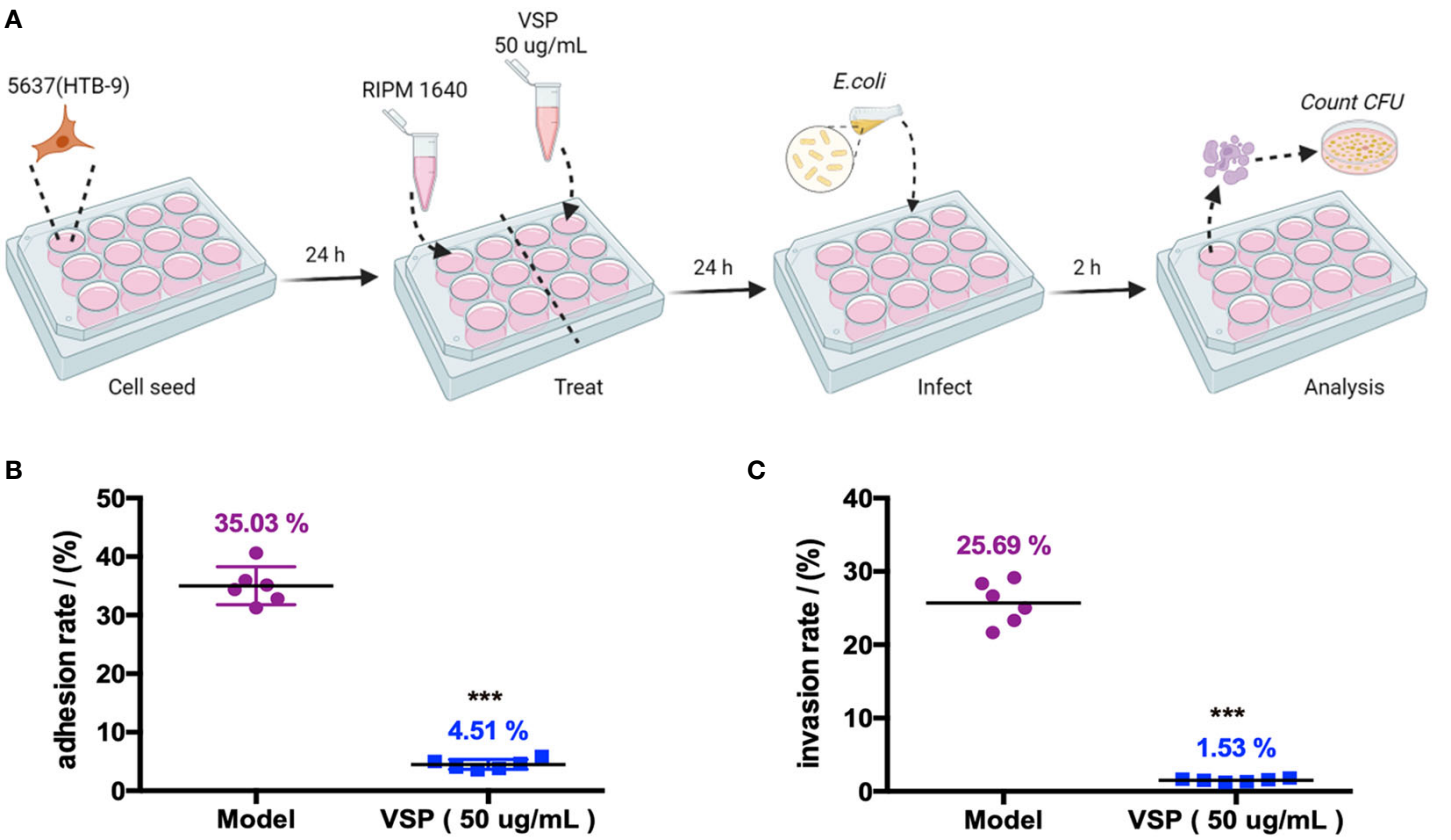
VSP inhibited the adhesion and invasion of UPEC CFT073 on the 5637 (HTB-9) cells. **(A)**, A scheme of the VSP administration and *E. coli* infection. **(B, C)**, The adhesion and invasion rates of UPEC CFT073 were assessed on MacConkey agar plates. Each symbol in this graph represents the value from one single group, and the geometric mean is shown as a horizontal line. *n*=6, compared with the model group: ^***^ *p* < 0.001, the results were determined by the Mann-Whitney test.

Adhesion and invasion are two key pathogenic steps in UPEC-induced UTIs. In this study, UPEC CFT073 infecting 5637 (HBT-9) cells was used to investigate the effects of VSP on bacterial adhesion and colonization. The results showed that UPEC CFT073 could quickly adhere to and invade bladder epithelial cells (BECs). After 2 hours of infection, the bacterial adhesion rate was 35.03%, and the invasion rate was 25.69%. After VSP intervention, the adhesion rate of CFT073 decreased to 4.51%. The number of bacteria that invaded the 5637 (HBT-9) cells was also significantly downregulated, and the invading CFUs were reduced to 1.53% of the total bacterial population (**Figure 2**). The results demonstrated that VSP significantly reduced the number of bacteria that adhered to and invaded bladder epithelial cells, indicating that VSP impairs the ability of UPEC to adhere and invade.

### 4.2 VSP reduces the expression of UPEC adhesion factors

**Figure 3:**
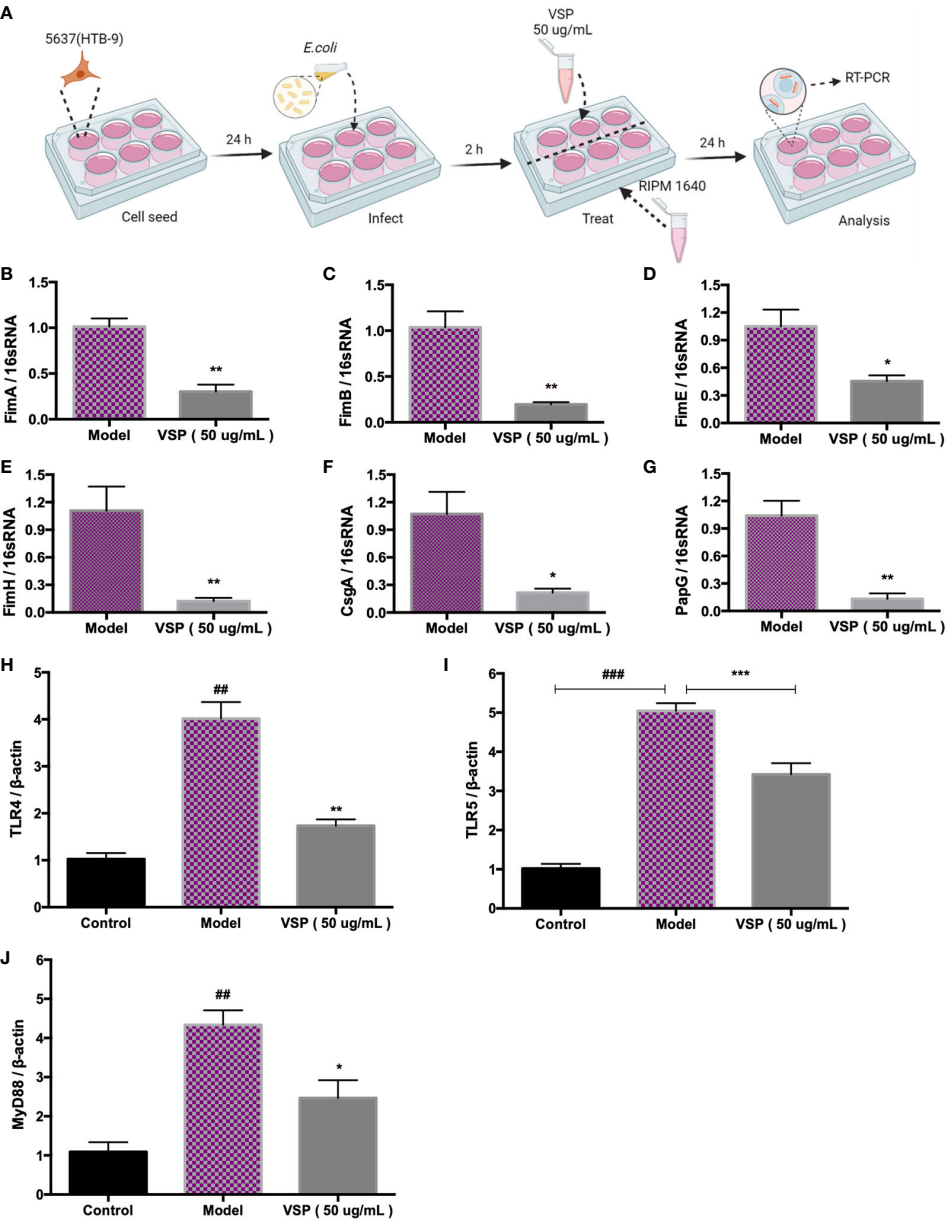
VSP inhibited the adhesion of UPEC CFT073. **(A)**, A scheme of the *E. coli* infection and VSP administration. **(B-G)**, The relative transcript levels of UPEC CFT073 adhesion fibronectin FimA, FimB, FimE, FimH, CsgA, and PapG after VPS intervention, were assessed by RT-PCR with 16S RNA as the internal control. **(H-J)**, The relative transcript levels of TLR4, TLR5 and MyD88 in 5637 (HTB-9) cells after the VPS intervention, were assessed by RT-PCR with β-actin as the internal control. *n*=4, compared with the control group: ^##^ *p* < 0.01. Compared with the model group: ^*^ *p* < 0.05, ^**^ *p* < 0.01, the results were determined by one-way ANOVA followed by Dunnett’s *post hoc* test. ^###^ *p* < 0.001, ***p < 0.001.

Fimbriae are complex surface structures that mediate the adherence of bacteria to host epithelial receptors, which are essential for UPEC to adhere to urothelial cells. To study the effects of VSP on UPEC fimbriae-related adhesion genes, we infected bladder epithelial cells with UPEC CFT073 and then treated them with VSP for 24 hours (**Figure 3A**). The results showed that two hours after the UPEC CFT073 infection of 5637 (HTB-9) cells, the expression levels of FimA, FimB, FimE, FimH, PapG and CsgA located at the distal end of the fimbriae were significantly increased. After the VSP intervention, the expression levels of the six adhesins were significantly downregulated compared with that in the model control group (**Figures 3B–G**). These results suggest that VSP effectively reduced the contact of UPEC with the surface of urothelial cells by inhibiting the expression of I, P and Curli pili tip adhesion proteins.

### 4.3 VSP downregulates the mRNA levels of TLRs in BECs

TLRs control the mucosal defence against pathogens. Toll-like receptor 4 (TLR4) and Toll-like receptor 5 (TLR5) identify P fimbriae and flagellins of bacteria, respectively. They are important for the inflammatory response and bacterial clearance. To verify the intervention effect of VSP on TLRs, we examined the expression of TLR4 and TLR5 in 5637 (HTB-9) bladder epithelial cells after infection with UPEC. The results showed that after 24 hours of infection with UPEC CFT073, the expression of TLR4, TLR5 and myeloid differentiation Factor 88 (MyD88) mRNA in the 5637 (HTB-9) bladder epithelial cells increased significantly. The mRNA expression of TLR4, TLR5 and MyD88 in the cells after the VSP treatment group showed a significant downward trend compared with the model group (**Figures 3H–J**).

### 4.4 VSP reduces the motility of bacteria

**Figure 4:**
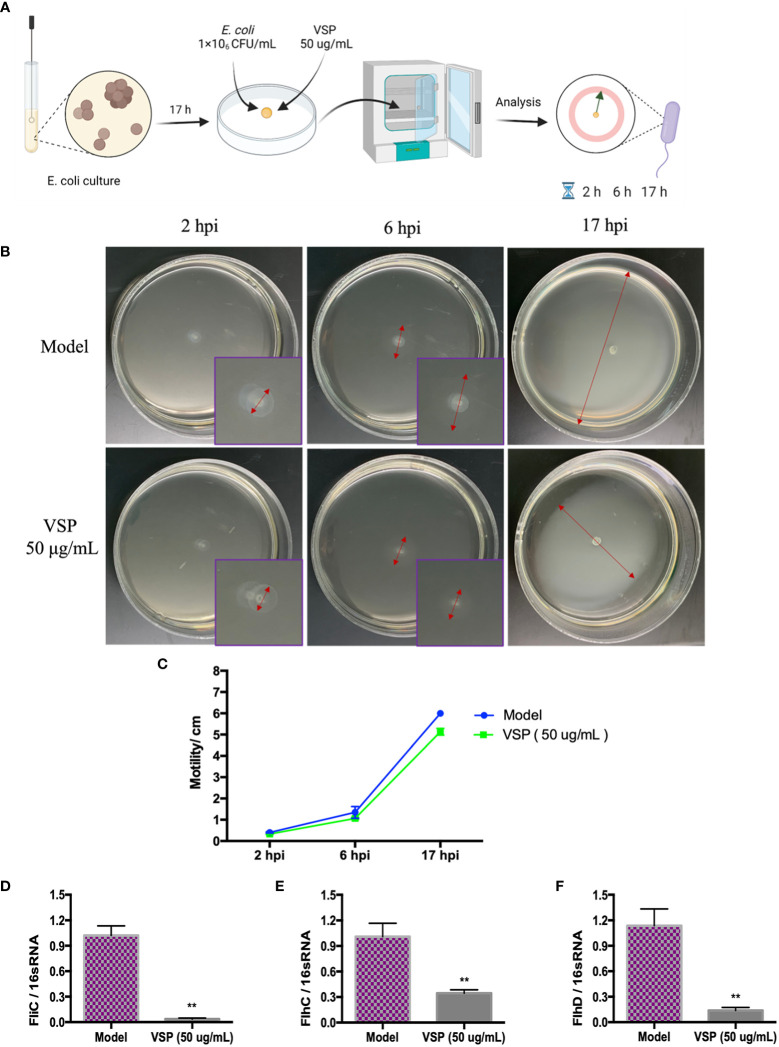
VSP inhibited the motility of UPEC CFT073. **(A)**, a scheme of the (*E*) *coli* infection and VSP administration. **(B)**, The motility of UPEC CFT073 in soft agar. The symbol in this graph represents the swimming motility diameter of UPEC CFT073. **(C)**, The zone of swarming was measured after 2 hours, 6 hours, and 17 hours. Each data point in the line graph indicates the means of six replicates. **(D-F)**, The relative transcript levels of fliC, FlhC and flhD after the VPS intervention, assessed by RT-PCR with 16S RNA as the internal control. *n*=4, compared with the control group. Compared with the model group: ^**^ *p* < 0.01, the results were determined by one-way ANOVA followed by Dunnett’s *post hoc* test.

Flagella and fimbriae are important in the colonization of the urinary tract by UPEC. Fimbriae facilitate adhesion to mucosal cells and promote bacterial persistence in the urinary tract, while flagella propel bacteria through urine along mucous layers during ascension to the upper urinary tract. In this experiment, we investigated whether the effect of VSP on locomotion impacted the pathogenesis of UPEC by detecting the mean diameter of UPEC CFT073 and the locomotor-regulated genes expressed by flagella. We found that after being inoculated on the soft agar plate, bacteria quickly multiplied and spread in the semi-solid agar solution, accompanied by the turbidity of the matrix, which showed the adaptability and diffusion of bacteria. The diameters of UPEC CFT073 movement in the model control group and the VSP intervention group were detected 2, 6, and 17 hours after infection (**Figure 4A**). We found that the bacteria in the VSP intervention group showed lower motility (**Figures 4B, C**). The mRNA expression levels of the UPEC flagellar control genes fliC, flhC, and flhD were also analysed using RT-PCR. These assays revealed that the levels of the three genes correlate well with the motility of UPEC CFT073. Expressing more flagellar genes results in more motility. It is worth noting that VSP (50 μg/mL) significantly inhibited the expression of UPEC flagellar control genes (fliC, flhC, and flhD), which significantly differed from the model control group (**Figures 4D–F**). The results suggest that VSP could reduce UPEC colonization in the urethra by downregulating flagella-mediated motility.

### 4.5 VSP reduces the bacterial titers in the bladder of the UTI mouse model

**Figure 5:**
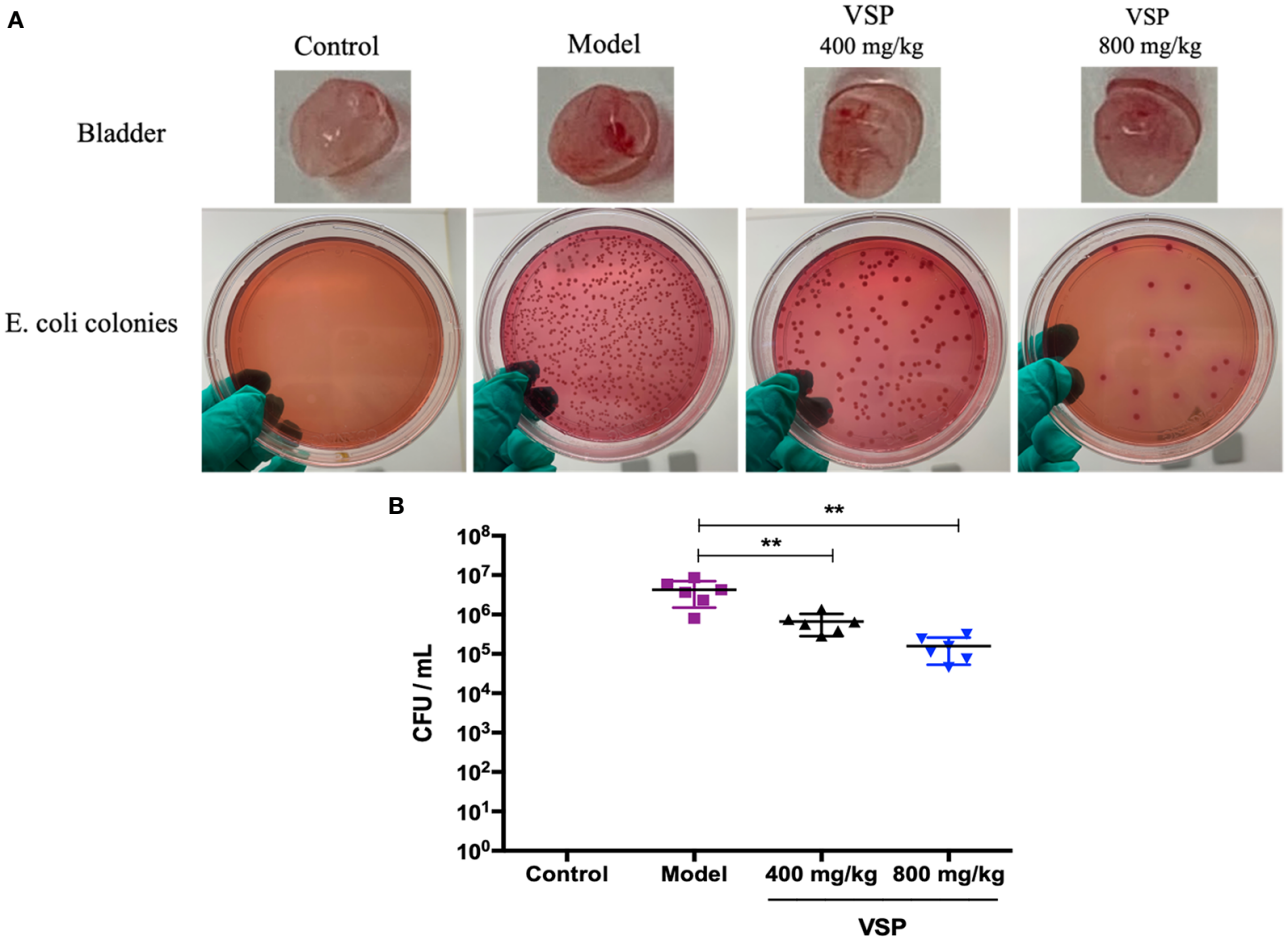
VSP reduced bacteria in the mouse bladders. **(A)**, The bladders of the mice were homogenized, diluted, and plated on MacConkey agar to determine bacterial counts. **(B)**, The results are presented as log_10_ CFU/mL. Each data point represents a sample from an individual mouse, and the horizontal line indicates the median. *n*=6 mice per group, compared with the model group: ^**^ *p* < 0.01, the results were determined by the Mann-Whitney test.

To further observe the intervening impacts of VSP on UPEC in murine urethral colonization, we assessed host colonization using the murine model of UPEC CFT073 infection. Six hours after the CFT073 infection, a large number of bacteria were detected in the bladder tissue of the C3H/HeN mice. With the 3 days of preventive administration of VSP, the bacterial titers of the bladder tissue in the 800 and 400 mg/kg groups were decreased, which was significantly different from that of the model group (**Figure 5**). *In vivo* investigations indicated that a 3-day pretreatment with PSV could reduce bacterial bladder colonization.

### 4.6 VSP inhibited the expression of uroplakins in the bladder of the UTI mouse model

**Figure 6:**
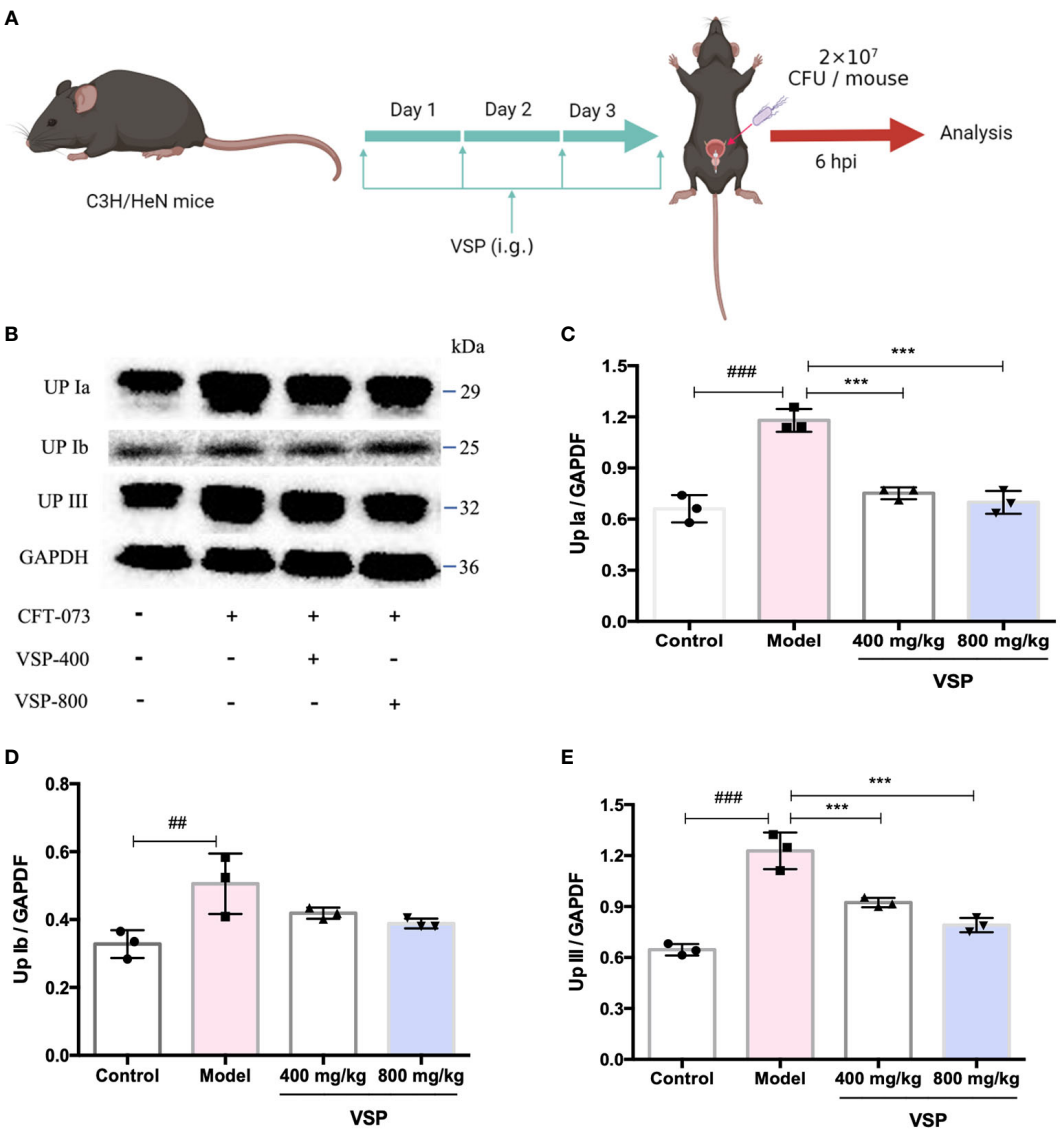
VSP inhibited the protein levels of uroplakins in the mouse bladder. **(A)**, a Scheme of the VSP administration and *(E) coli* infection. **(B)**, The Western blot results of UP Ia, UP Ib and UP III. **(C-E)** Quantitative analysis of UP Ia, UP Ib and UP III protein bands. *n*=3 mice per group, compared with the control group: ^##^ *p* < 0.01, ^###^ *p* < 0.001; compared with the model group: ^***^ *p* < 0.001. The results were determined by one-way ANOVA followed by Dunnett’s *post hoc* test.

**Figure 7:**
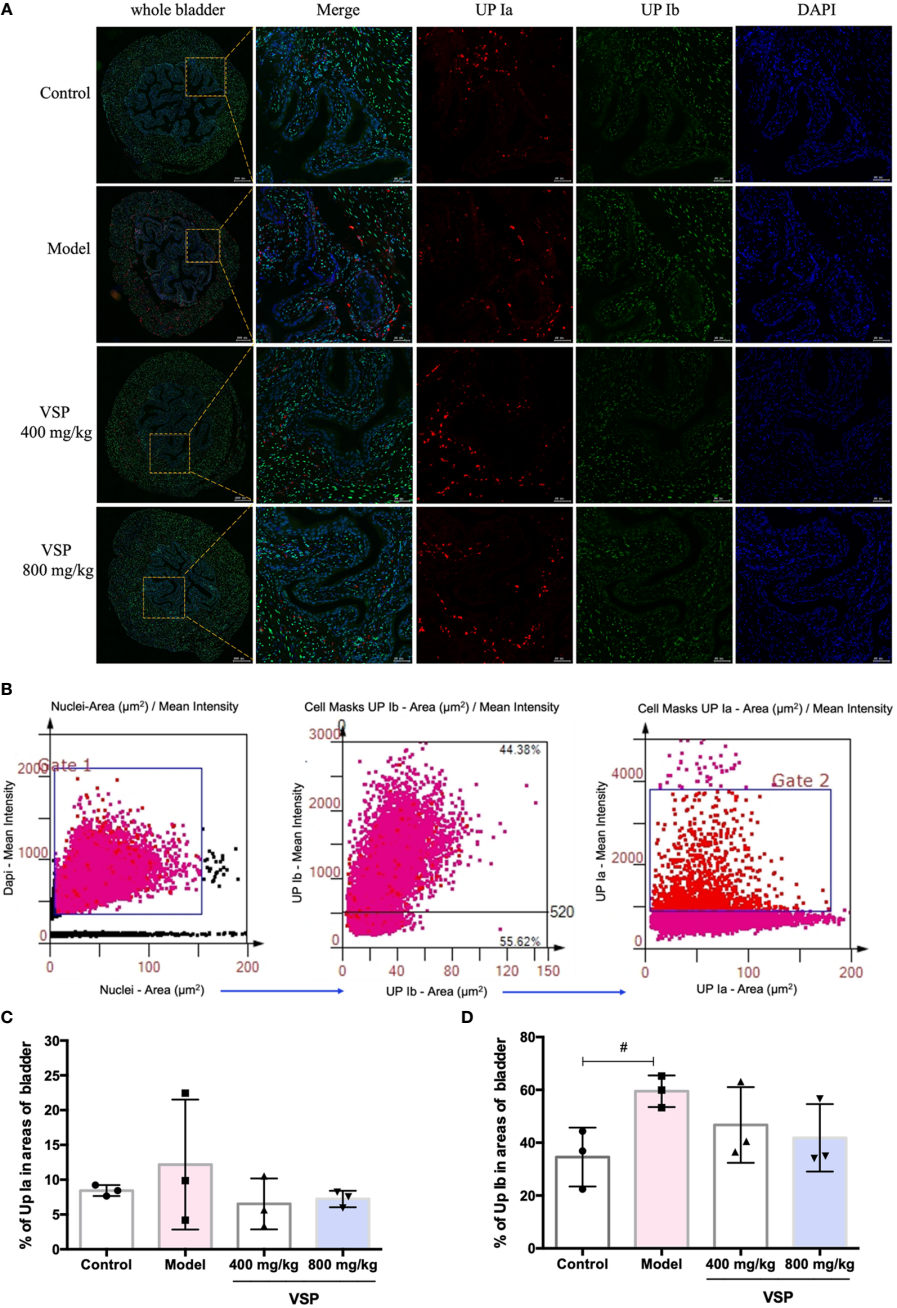
VSP inhibited the levels of UP Ia and UP Ib in the mouse bladder. **(A)**, Representative costained images with UP Ia, UP Ib, and DAPI captured the bladder tissue of the UPEC CFT073-infected C3H/HeN mice. Yellow rectangle: higher magnification view of the selected area (parallel sections, the green signals represent UP Ia, the red signals represent UP Ib, the blue signals represent DAPI, scale bar = 50 μm); **(B)**, Representative scatterplots for the UPIa and UPIb expression levels from the bladder tissue of the C3H/HeN mice infected with UPEC CFT073. **(C, D)**, Statistical analysis of the UPIa- and UPIb-positive cells in the entire cross-sectional tissue region of the mouse bladder. Quantitatively, the percentage of cells expressing UPIa (UPIa/DAPI) and UPIb (UPIb/DAPI). *n*=3 mice per group, compared with the control group: ^#^ *p* < 0.05. The results were determined by Wilcoxon rank-sum test.

**Figure 8:**
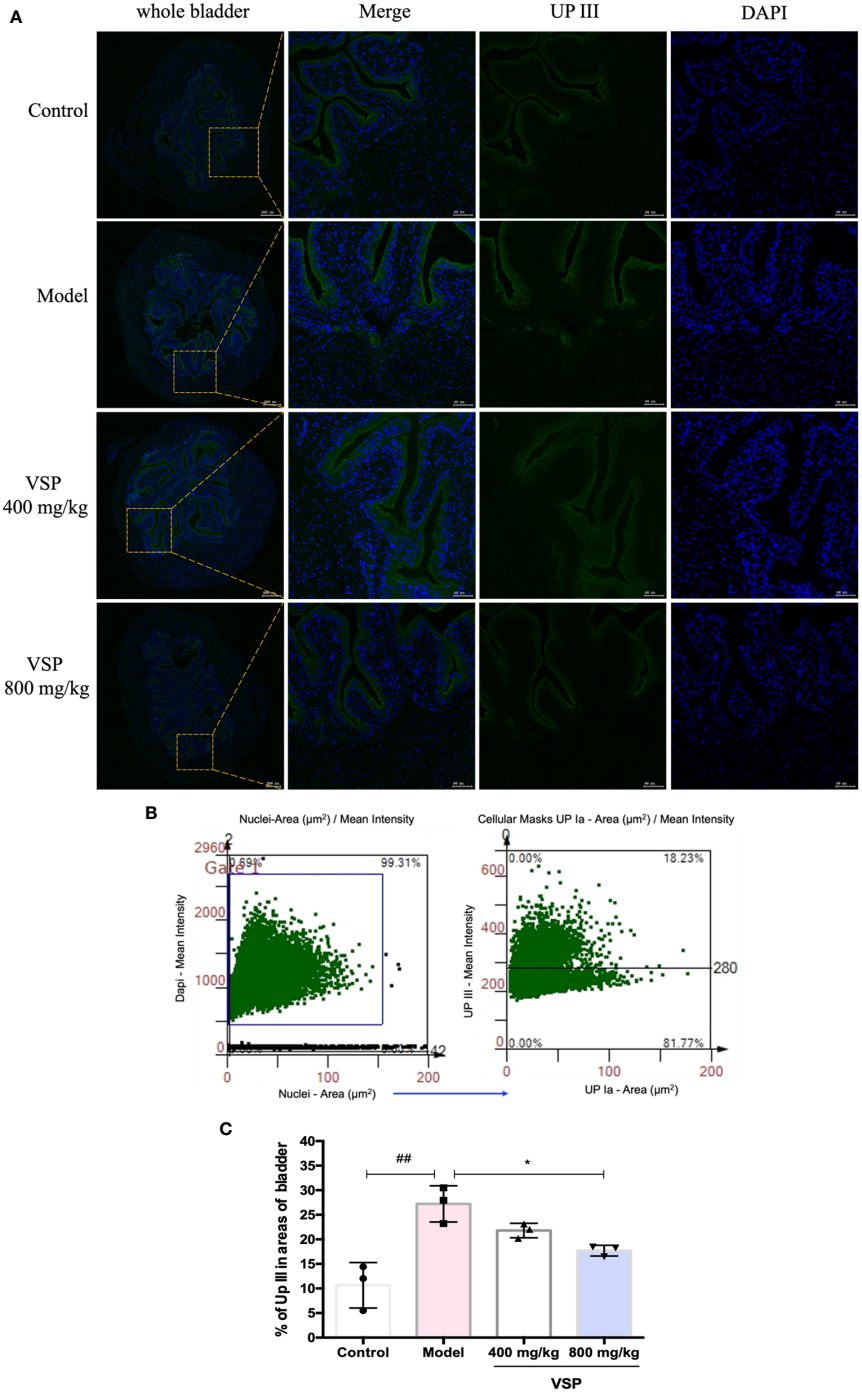
VSP inhibited the protein level of UP III in the mouse bladder. **(A)**, Representative co-stained images with UP III and DAPI captured the bladder tissue of the UPEC CFT073-infected C3H/HeN mice. Yellow rectangle: higher magnification view of the selected area (parallel sections, the green signals represent UP III, the blue signals represent DAPI, scale bar = 50 μm). **(B)**, Representative scatterplots for UPIII expression from the bladder tissue of the C3H/HeN mice infected with UPEC CFT073. **(C)**, Statistical analysis of the UPIII-positive cells in the entire cross-sectional tissue region of the mouse bladder. Quantitatively, the percentage of cells expressing UPIII (UPIIII/DAPI). *n*=3 mice per group, compared with the control group: ^##^ *p* < 0.01; compared with the model group: ^*^p < 0.05. The results were determined by Wilcoxon rank-sum test.

Considering that UPEC binds and invades the urinary system, various hosts may show differences in their epithelial structure and surface receptors. We assessed the expression of uroplakins using the UPEC CFT073-infected mouse model (**Figure 6A**). We found that after six hours of the UPEC CFT073 infection, uroplakins (UP Ia, UP Ib and UP III) were upregulated in the mice bladders. Western blotting results revealed that after the treatment with VSP (800 mg/kg, 400 mg/kg), the expression levels of UP Ia and UP III in the bladder of the mice were significantly downregulated (**Figures 6B–E**). Immunofluorescence staining results showed that UP Ia was highly expressed in the submucosa and muscle layer of the bladder tissues (**Figure 7A**), and UP III was highly expressed in the upper mucosa of the bladder tissues (**Figure 8A**). After treatment with VSP (800 mg/kg), the expression levels of UP III in the bladder of the mice was significantly downregulated (**Figures 8B, C**). UP Ia and UP Ib showed a downward trend, which was not statistically different from the model group (**Figures 7B–D**). The changes in uroplakins in the mice bladder tissues were similar in the western blot and immunofluorescence results. Our results suggested that reducing the bacterial binding to the host surface receptors was one of the pathways by which VSP protects bladder epithelial cells from UPEC infection.

## 5 Discussion

UPEC adhesion and colonization are the initial mechanisms that cause UTIs. UPEC adhesion to urothelial cells and the subsequent tissue colonization are necessary for the bacteria to resist urine flow and establish further infections. The rapid colonization of UPEC in the urinary tract helps the bacteria evade immune attacks and thus maintains their invasion in tissues (Anderson et al., 2003; Brannon et al., 2020). In this study, to explore the effects of VSP on bacterial adhesion and invasion, UPEC CFT073 was used to infect urethral epithelial cells and C3H/HeN mice to establish UTI models.

Our previous research showed that VSP reduced the number of bacteria which successfully invaded the urine, bladder and kidney of the UTI model mice. However, in our previous *in vitro* study, VSP showed no antibacterial effect against UPEC CFT073 (Mao et al., 2020), which indicates that the therapeutic effect of VSP in UTIs may not depend on its direct killing effect on UPEC. Combined with the research of Fim H and PapG inhibitors, we speculate that the mechanism of VSP in the treatment of UTIs may lie in inhibiting bacterial adhesion and invasion, and intervening in the process of bacterial and host infection.

First, this study examined the effect of VSP on the adhesion and invasion of bladder urothelial cells by the UPEC CFT073 strain. The results showed that the VSP intervention significantly reduced the number of bacteria that successfully adhered to the 5637 bladder epithelial cells (HBT-9). In addition, the ability of UPEC to invade the urinary tract cells was significantly blocked. This shows that VSP weakened the adhesion and invasion ability of UPEC. To explore the mechanism by which VSP inhibits the adhesion and invasion of UPEC, we observed various virulence factors of UPEC. Virulence factors, such as I fimbria, P fimbria, curled fimbria and flagella, are attractive candidates for the development of new drugs and vaccines (O’Brien et al., 2016; Sharma et al., 2021).

Fimbriae bind to receptors on the surface of urothelial cells and mediate the contact of bacteria with various host cells to achieve invasion and reproduction of the urinary tract (Beydokhti et al., 2019; Day et al., 2021). The recognized UPEC reference strain CFT073 encodes 13 distinct fimbrial gene clusters, including type 1, P, F1C, Dr, Auf, S, and M fimbriae. These gene clusters, or operons, encode all of the genes necessary for the assembly of the fimbriae, including structural subunits as well as chaperone and usher proteins that aid in their secretion (Nielubowicz and Mobley, 2010; Klein and Hultgren, 2020). The pathogenic mechanism of UPEC begins with the adhesion of adhesins on the distal end of fimbriae to the host (Luna-Pineda et al., 2016). These adhesins interact with different receptors (α-D-mannosylated proteins, glycosphingolipids, neuraminic acids, lactosylceramides, decay-accelerating factors, and matrix proteins) located on the urethral cell membrane (Antaão et al., 2009; Lüthje and Brauner, 2014; Luna-Pineda et al., 2019). The recombinases (fimH, fimA, fimB, fimE) of UPEC type I fimbriae interact with uroplakins and other host proteins that contain mannosidaes, leading to an infiltration process that allows UPEC to avoid the flow of urine, antibodies in urine, bactericidal molecules, and antibiotic activity (Mulvey et al., 1998; McLellan et al., 2021). The P fimbriae of UPEC enable colonization and inflammatory responses by binding papG adhesion to glycolipids and TLR4 in host cells (Fischer et al., 2006; Huang et al., 2020; Lane et al., 2007a). Curli fimbriae, mainly composed of csgA protein monomers, are associated with acute cystitis and pyelonephritis (Hung et al., 2014; Lim et al., 2014; Beydokhti et al., 2019). These fimbriae are widely distributed in clinical strains of UPEC, and by binding to receptors on the luminal surface of urothelial cells, they mediate bacterial contact with various host cells, achieve urinary tract invasion and clonality, and cause UTIs.

This research tested the effects of VSP on the expression levels of fibronectin genes mediated by UPEC CFT073 I fimbria (FimH, FimA, FimB, FimE), P fimbria (PapG) and curled fimbria (CsgA). The results showed that the mRNA expression levels of the six fibronectins were significantly reduced after the VSP intervention, indicating that VSP effectively inhibited the contact between bacteria and the surface of the urothelial cells by controlling the expression of adhesion factors. We hypothesize that the reduced adherence and invasion ability of UPEC was caused by the decreased expression of fimbriae, such as type 1 and P fimbriae.

As the first cellular defence against bacterial infection, the urinary tract epithelium expresses a variety of pattern recognition receptors, and the pattern recognition receptors involved in recognizing UPEC infection are mainly TLRs. And in humans, the TLRs that contribute to the recognition of UPECs by the urinary tract are mainly TLR4 and TLR5. TLR4 signalling can be induced by multiple mechanisms, including a P fimbriae-dependent and lipopolysaccharide-independent pathway (Hedlund et al., 1999; Frendeus et al., 2001; Klarström Engström et al., 2019) and a lipopolysaccharide-dependent and type 1 fimbriae-dependent pathway (Hedlund et al., 2001; Klein and Hultgren, 2020). In addition, FimH can directly interact with TLR4 (Ashkar et al., 2008; Mossman et al., 2008; Chevalier et al., 2021). Regardless of the source of stimulation, TLR4-mediated signalling results in the activation of NF-κB and the expression of pro-inflammatory genes, such as interleukin-6 (IL-6) and interleukin-8 (IL-8) (Shaukat et al., 2021). TLR5 recognizes flagella, resulting in the production of pro-inflammatory factors (Hayashi et al., 2001; Andersen-Nissen et al., 2007; Akahoshi and Bevins, 2022). All known TLRs signal through the adaptor protein MyD88 except TLR3 (Björkbacka et al., 2004). The TLR4 response to type 1 fimbriated UPEC occurs through MyD88. The impaired antibacterial defence in Myd88-null mice suggests that MyD88 is required for neutrophil activation and bacterial killing in the urinary tract mucosa (Ciesielska et al., 2021). In addition, the TLR5 response to the flagellin of UPEC occurred through MyD88. Studies have shown that mice challenged with bacterial flagellin rapidly produced systemic IL-6, whereas MyD88-null mice did not respond to flagellin (Tahoun et al., 2017; Lei et al., 2021). The fimbriae and flagellin-specific adaptor protein responses were shown to influence the quality of the host response and the outcome of infection (Fischer et al., 2006).

Our results showed that the expression levels of TLR4, TLR5, and MyD88 mRNA in the bladder epithelial cells were upregulated in response to the bacterial infection. And the expression levels of TLR4, TLR5, and MyD88 were downregulated significantly after the VSP intervention. The urothelium is an anatomical barrier to the innate immune response, which expresses TLRs with the capacity to recognize pathogen-associated molecular patterns. The involvement of TLRs can lead to uroepithelial cell activation and the production of inflammatory mediators. These results indicate that VSP reduces bacterial invasion and the release of inflammatory factors by inhibiting the expression of Toll-like receptors, thereby inhibiting bladder tissue damage.

Motility, an important factor in the pathogenicity of UTI pathogens, is mediated by flagella. The flagellum is the most important motor organ of UPECs, which serve as a power device for adhesion, secrete virulence factors, and plays an important role in biofilm formation (Mitra et al., 2013; Terlizzi et al., 2017). Bacterial flagella are long helical surface appendages composed of fliC-encoded polymeric subunits of flagellin. The mutation of fliC in UPEC results in the loss of flagella and motility. High fliC expression is consistent with the bacterial ascent to the upper urinary tract, suggesting that motility is involved in the migration of infection from the bladder to the kidney (An et al., 2021; Lane et al., 2007b). The flhDC operon, composed of flhC and flfD genes, is the master regulator of flagella production. Products encoded by the flhC and flfD genes constitute the FlhD4-FlhC2 heterohexameric structure transcription activator protein. The flhDC of the pili master regulator can be assessed by detecting the expression levels of flhC and flfD (Wang et al., 2006; Li et al., 2021; Thai et al., 2021). Swarming motility is a flagella-dependent form of bacterial motility that facilitates bacterial migration on viscous substrates. Swarming motility regulates gene expression and controls many physiological activities, including virulence factor production, movement and biofilm formation (Ranfaing et al., 2018; Li et al., 2019).

The soft agar plate method was used to explore the effects of VSP on flagella-mediated swarming motility. The results showed that VSP inhibited the motility of UPEC CFT073. To explore the mechanism by which VSP regulates motility, we examimarina ortigueraned candidate genes that might affect these functions. fliC encodes bacterial flagella. The promoter region for class I flagellar genes, flhC and flhD, initiates the complex cascade of gene expression leading to flagellar expression (Cooper et al., 2012). Our results demonstrate that VSP inhibited the expression of fliC, flhC and flhD, which suggests that VSP could downregulate the motility mediated by flagella and thereby prevent upper urinary tract infections caused by UPEC.

*In vivo*, six hours after the UPEC CFT073 infection, a large number of bacteria could be detected in the bladder tissue of the mice. In the VSP pre-administered treatment group, the number of colonies in the bladder tissues of the mice were significantly reduced. To further understand the mechanism by which VSP inhibits bacterial adhesion and invasion, this study observed the expression of uroplakins. The four major uroplakins (UPIa, UPIa, UPII and UPIII) are expressed in urothelial cells (Scharf et al., 2019; Day et al., 2021). UPIa and UPIb can specifically bind to UPEC type 1 fimbriae, allowing UPEC to gain an initial foothold in the urinary tract (Wu et al., 1996; Mohanty et al., 2018). UPIII is the only uroplakin with a cytoplasmic domain and is sensitive to UPEC-induced urothelial cell death (Thumbikat et al., 2009; Szymańska et al., 2021). UPIa and UPIb form heterodimers with UPII and UPIII, respectively, which assemble into higher-order complexes. The uroplakin complexes help strengthen urothelial cell stability, maintain a permeable barrier, and prevent bladder damage.

Immunofluorescence was employed to detect the position and intensity of uroplakins in the bladder tissue of the UTI mouse model. We found that VSP reduced the fluorescence intensity of urine plaque protein in the UTI model mice. Western blotting was employed to detect the expression of uroplakins in the bladder tissue of the UTI model mice. The results showed that 6 hours after the UPEC CFT073 strain infection of the C3H mice, uroplakin proteins were highly expressed in the bladder tissues. After VSP intervention, the expression levels of UP Ia and UP III in the bladder tissue of the UTI model mice were significantly downregulated. These results suggest that VSP reduced adhesion and invasion by inhibiting the expression of uroplakins.

In summary, VSP reduced the adhesion, colonization, and motility of UPEC CFT073 and prevented pathogenic bacteria from internalizing into the urinary tract mucosal cells, thereby inhibiting bacterial infection. Our results indicate that VSP effectively inhibited UPEC infection and has the potential to be developed into a new alternative antibiotic treatment for UTIs. The findings of our research support the potential use of VSP in the treatment of UTIs. However, more detailed clinical studies on humans should be performed.

## Data availability statement

The original contributions presented in the study are included in the article/supplementary material. Further inquiries can be directed to the corresponding authors.

## Ethics statement

Animal experimentation and the corresponding protocol (No. 2019-0085) were approved by the Animal Ethics Committee of the Institute of Chinese Materia Medica China Academy of Chinese Medical Sciences.

## Author contributions

XC and RY designed this research; HZ provided the VSP; RY, XM, YX, LB, YB, SG, YS and YG carried out the experiments; RY analyzed the results and wrote the manuscript. XC and LH revised the manuscript. All authors contributed to the article and approved the submitted version.

## Funding

This work was supported by the National Science and Technology Major Project (no. 2019ZX09721001-005-001).

## Conflict of interest

Author XM was employed by company Guangzhou Baiyunshan Xingqun Pharmaceutical Co., Ltd.

The remaining authors declare that the research was conducted in the absence of any commercial or financial relationships that could be construed as a potential conflict of interest.

## Publisher’s note

All claims expressed in this article are solely those of the authors and do not necessarily represent those of their affiliated organizations, or those of the publisher, the editors and the reviewers. Any product that may be evaluated in this article, or claim that may be made by its manufacturer, is not guaranteed or endorsed by the publisher.

## Abbreviations

UTIs, urinary tract infections; UPEC, uropathogenic *Escherichia coli*; *E coli*, *Escherichia coli*; VSP, polysaccharide extract from Vaccaria segetalis seeds; BECs, bladder epithelial cells; CFU, colony-forming unit; TLR4, Toll-like receptor 4; TLR5, Toll-like receptor 5; MyD88, myeloid differentiation Factor 88; UP Ia, Uroplakin Ia; UP Ib, Uroplakin Ib; UP III, Uroplakin III.
